# Cerebral Activity Changes in Different Traditional Chinese Medicine Patterns of Psychogenic Erectile Dysfunction Patients

**DOI:** 10.1155/2015/503536

**Published:** 2015-06-09

**Authors:** Qi Liu, Peihai Zhang, Junjie Pan, Zhengjie Li, Jixin Liu, Guangsen Li, Wei Qin, Yaodong You, Xujun Yu, Jinbo Sun, Minghao Dong, Qiyong Gong, Jun Guo, Degui Chang

**Affiliations:** ^1^The 3rd Teaching Hospital, Chengdu University of Traditional Chinese Medicine, Chengdu, Sichuan 610075, China; ^2^The Urology and Andrology Department, Sichuan Integrative Medicine Hospital, Chengdu, Sichuan 610041, China; ^3^The Urology and Andrology Department, The 1st Teaching Hospital, Chengdu University of Traditional Chinese Medicine, Chengdu, Sichuan 610072, China; ^4^The Urology and Andrology Department, Traditional Chinese Medicine Hospital of Meishan City, Meishan, Sichuan 620010, China; ^5^Life Sciences Research Center, School of Life Sciences and Technology, Xidian University, Xi'an, Shaanxi 710071, China; ^6^The Andrology Department, The 2nd Teaching Hospital, Chengdu University of Traditional Chinese Medicine, Chengdu, Sichuan 610041, China; ^7^Department of Radiology, The Center for Medical Imaging, Huaxi MR Research Center, West China Hospital, Sichuan University, Chengdu, Sichuan 610041, China; ^8^The Andrology Department, Xiyuan Hospital of China Academy of Chinese Medical Science, Beijing 100091, China

## Abstract

*Background.* Pattern differentiation is the foundation of traditional Chinese medicine (TCM) treatment for erectile dysfunction (ED). This study aims to investigate the differences in cerebral activity in ED patients with different TCM patterns.* Methods.* 27 psychogenic ED patients and 27 healthy subjects (HS) were enrolled in this study. Each participant underwent an fMRI scan in resting state. The fractional amplitude of low-frequency fluctuation (fALFF) was used to detect the brain activity changes in ED patients with different patterns.* Results.* Compared to HS, ED patients showed an increased cerebral activity in bilateral cerebellum, insula, globus pallidus, parahippocampal gyrus, orbitofrontal cortex (OFC), and middle cingulate cortex (MCC). Compared to the patients with liver-qi stagnation and spleen deficiency pattern (LSSDP), the patients with kidney-yang deficiency pattern (KDP) showed an increased activity in bilateral brainstem, cerebellum, hippocampus, and the right insula, thalamus, MCC, and a decreased activity in bilateral putamen, medial frontal gyrus, temporal pole, and the right caudate nucleus, OFC, anterior cingulate cortex, and posterior cingulate cortex (*P* < 0.005).* Conclusions.* The ED patients with different TCM patterns showed different brain activities. The differences in cerebral activity between LSSDP and KDP were mainly in the emotion-related regions, including prefrontal cortex and cingulated cortex.

## 1. Introduction

Erectile dysfunction (ED), defined as the consistent inability to attain or maintain penile erection sufficient for satisfactory sexual performance [1], has become a global health issue with a high prevalence [2, 3] and considerable impact on the quality of life (QoL) of sufferers and their partners [4, 5]. In addition, ED may share a common pathologic mechanism with cardiovascular diseases [6–8], metabolic syndromes [9], and other endocrine disorders [10, 11]. ED is generally categorized as organic, psychogenic, or mixed. Psychogenic ED is diagnosed when physical factors are excluded. Patient has risk factors and history for psychogenic ED and has normal androgen status and normal findings on penile duplex Doppler ultrasonography (DUS) [12, 13]. Although organic factors were responsible for the most of the ED cases, psychological factors contribute to and worsen the situation afterwards in almost every organically caused ED case [14].

Despite the advances in clinical and basic researches which have led to several new options, the ideal treatment of ED has not been identified [15]. TCM has been used to treat sexual dysfunction such as ED in China for more than 2000 years. A number of studies showed that TCM treatment could significantly improve the quality of erection and sexual activity of ED patients [16–20] and that correct pattern differentiation (“Bian Zheng”) was the prerequisite for achieving the hoped-for efficacy of TCM for treating ED. Pattern differentiation is one of the essential characters of TCM. It means analyzing and judging the data obtained from the four diagnostic methods (inspection, auscultation and olfaction, inquiry, and pulse-taking and palpation) so as to differentiate the nature, location, and cause of disease. So pattern differentiation is the premise and foundation of treatment. However, the pattern differentiation of TCM is mainly based on subjective symptoms and signs, such as tongue condition and pulse condition, and the collection and analysis of those symptoms and signs nearly depend on the clinical experience of doctors. This phenomenon significantly affects the veracity of pattern differentiation and the curative effect of TCM further. So seeking more objective and intelligible evidence for pattern differentiation becomes an urgent task in TCM research and attracts increasing attention.

Normal erectile function is an integrated response under the control of the central nervous system (CNS) and involves the supraspinal centers, the spinal cord, and peripheral nerves. In the last decade, using functional neuroimaging techniques, people found that many brain regions are involved in the supraspinal control of penile erection including the cingulate cortex (CC), insula, orbitofrontal cortex, caudate nucleus, putamen, thalamus, and hypothalamus [21–25]. Furthermore, recent studies [26] indicated that, compared to HS, ED patients showed significant and extended activation in multiple brain regions including the CC, frontal mesial, and frontal basal cortex. Our previous study [27] showed increased fractional anisotropy (FA) values, reduced mean diffusivity (MD) values, and reduced axial diffusivity (AD) values in multiple white matter tracts including the corpus callosum (genu, body, and splenium), corticospinal tract, internal capsule, corona radiata, external capsule, and superior longitudinal fasciculus. Whether there are significant differences in cerebral activities among ED patients with different TCM patterns and whether the brain function changes can be used as potential evidences for pattern differentiation remain uncovered and are worthy of investigation.

By functional magnetic resonance imaging (fMRI), this study aims to (a) investigate the brain activity changes of psychogenic ED patients by comparing with those of HS and (b) explore the differences in cerebral activity between two TCM patterns of psychogenic ED.

## 2. Materials and Methods

### 2.1. Participant

#### 2.1.1. Psychogenic ED Patient

ED patients were recruited from the Outpatient Department in The 1st and 2nd Teaching Hospital of Chengdu University of Traditional Chinese Medicine from May 2012 to December 2012. Twenty-seven ED patients were enrolled in this study after undergoing (1) a detailed history taking, including psychosocial history (including the patient's assessment of his own sexual performance and his general attitude and knowledge about sex), medical history, relevant drug history (including alcohol, tobacco, or illicit drug use), and surgical disorder; (2) a careful physical examination, especially the urology and andrology examination; (3) a basic laboratory test which included a penile duplex Doppler ultrasonography (DUS), a nocturnal penile tumescence (NPT) test, as well as routine blood examinations, thyroid-stimulating hormone level, prostate-specific antigen, and the serum sexual hormone status (free/total testosterone, sexual hormone binding globulin, follicle-stimulating hormone, luteinizing hormone, estrogen, and prolactin); and (4) a psychophysical status evaluation by 2 separate psychologists.

Inclusion criteria were (1) 18–45 of age, (2) being right-handed, (3) having been in impotence for more than six months according to National Institutes of Health criteria [1], (4) being diagnosed as psychological ED, and (5) being in a stable heterosexual relationship for at least one year.

Exclusion criteria were (1) being diagnosed as organic ED or (2) having a history of urological surgery or head trauma with loss of consciousness or (3) having been using medication affecting sexual function for over two weeks before enrollment or (4) having suffered from serious psychiatric, neurological, cardiovascular, respiratory, gastrointestinal, or renal illnesses or (5) being drug or alcohol users and smokers.

#### 2.1.2. Healthy Subject

Twenty-seven right-handed and age-matched healthy subjects (HS) without urosexual symptoms or signs, psychiatric or neurologic disorders were enrolled in this study by advertisement. The same examinations including history taking and physical and laboratory tests were performed on all HS by andrology physician and psychologist.

### 2.2. Ethics Statement

This study was performed according to the principles of the Declaration of Helsinki. The study protocol was approved by the Ethics Committee of Chengdu University of Traditional Chinese Medicine. Written consent was obtained from all participants.

### 2.3. Symptom Assessment

The symptom severity was assessed by the International Index of Erectile Function (IIEF-5) [28], Quality of Erection Questionnaire (QEQ) [29, 30], the Erection Hardness Score (EHS) [31], and the self-esteem and relationship (SEAR) [32, 33]. Also, the Self-Rating Depression Scale (SDS) [34] and the Self-Rating Anxiety Scale (SAS) [35] were used to evaluate the emotional state of the participants.

### 2.4. TCM Pattern Differentiation

The pattern differentiation of ED patients was employed according to the* Guiding Principle of Clinical Study on New Traditional Chinese Medicine.* Details were in the supplementary material [36].

After careful pattern differentiation, 10 of the 27 ED patients were differentiated from liver-qi stagnation and spleen deficiency pattern (LSSDP) and 10 patients from kidney-yang deficiency pattern (KDP) and 3 patients from dampness-heat accumulation pattern and 2 patients from kidney-yin deficiency pattern and 2 patients from heart-spleen deficiency pattern.

### 2.5. fMRI Data Acquisition

Resting state fMRI scan was acquired by a 3.0T Siemens scanner (Allegra, Siemens Medical System, Erlangen, Germany) at the Huaxi MR Research Center, West China Hospital of Sichuan University, Chengdu, China. To minimize the head motion to diminish scanner noise, a standard birdcage head coil was used along with a restraining foam pad. The axial three-dimensional T1-weighted image was obtained with a spoiled gradient recall sequence (TR = 1900 ms; TE = 2.26 ms; flip angle: 9°; in-plane matrix resolution: 256 × 256; slices: 176; field of view: 256 mm; voxel size: 1 × 1 × 1 mm^3^). Functional images were acquired by using a gradient-echo echo planar imaging sequence (TR = 2000 ms; TE = 30 ms; flip angle: 9°; in-plane matrix resolution: 64 × 64; slices: 30; voxel size: 3.75 × 3.75 × 5 mm^3^). During scanning, they were asked to remain relaxed, to keep their eyes closed, and to remain still.

### 2.6. fMRI Data Preprocessing

The fMRI images were preprocessed with the SPM8 (http://www.fil.ion.ucl.ac.uk/spm). All of the subject's head movements were less than 1 mm maximum displacement in any direction of *x*, *y*, and *z* and less than 1° in any angular dimension. The preprocessing steps were as follows: to discard the first ten volumes, correct slice timing and head motion, normalize the corrected images to Montreal Neurological Institute space with a resampling resolution of 3 × 3 × 3 mm^3^, remove linear trend, and smooth with a Gaussian kernel of 6 mm full width at half maximum.

### 2.7. Data Analysis

#### 2.7.1. Clinical Variables

All the physiologic and psychological measures were analyzed with SPSS 17.0 software (SPSS Inc., Chicago, IL). All data are given as mean ± standard deviation (SD). Independent-samples *t*-test was used on numerical variables and two-sided test was applied on all available data. *P* value < 0.05 was considered statistically significant.

#### 2.7.2. Fractional Amplitude of Low-Frequency Fluctuation (fALFF) Analysis

After the above preprocessing, the fractional amplitude of low-frequency fluctuation (fALFF) analysis was carried out using the Analysis of Functional Neuroimaging (AFNI) software [37]. The procedure of the analysis was as follows: (1) transforming the time series for each voxel to a frequency domain without band-pass filtering; (2) calculating the square root at each frequency of the power spectrum; (3) acquiring the sum of amplitude across 0.01–0.08 Hz and dividing the sum by that across 0–0.25 Hz (entire frequency range). The fractional value was taken as fALFF [38]. Therefore, fALFF represents a ratio of the power of each frequency at the low-frequency range (0.01–0.08 Hz) to that of the entire frequency range (0–0.25 Hz).

The individual fALFF map was transformed to Z score by subtracting the global mean value and being divided by the standard deviation. Spatial smoothing was conducted on the Z maps with an isotropic Gaussian kernel (6 mm). The Z maps were transformed into the Talairach and Tournoux coordinates [39]. The further processing of group comparisons was performed on the Z maps to evaluate fALFF differences. The threshold for statistical significance was *P* < 0.005, using threshold-free cluster enhancement (TFCE) method [40, 41].

## 3. Results

### 3.1. Differences between Psychogenic ED Patients and HS

#### 3.1.1. Clinical Variables

Compared to HS, the ED patients showed significant decrease in the IIEF-5 score, EHS score, SEAR score, and QEQ score (*P* < 0.01) (Table 1).

#### 3.1.2. Brain Activity

**Figure 1:**
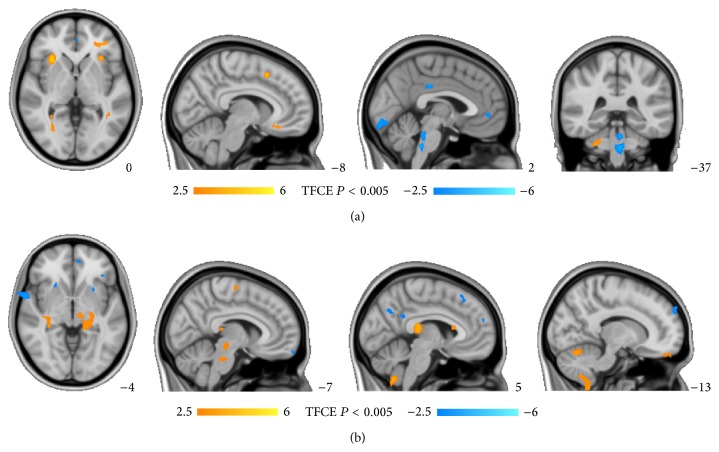
The differences in cerebral activities between psychogenic ED patients with different TCM patterns. (a) The differences in cerebral activities between psychogenic ED patients and HS. Compared to HS, psychogenic ED patients showed abnormal cerebral activity in brainstem, cerebellum, basal ganglia, and multiple limbic regions including insula, MCC, PFC, parahippocampal gyrus, and parietal, temporal lobes. (*P* < 0.005, a minimal cluster size of 50 voxels). (b) The differences in cerebral activities between ED patients with different TCM patterns. The brain regions associated with emotion modulation such as cerebellum, OFC, ACC, and MCC are the main different brain areas between psychogenic ED patients with kidney-yang deficiency pattern and liver-qi stagnation and spleen deficiency pattern (*P* < 0.005, a minimal cluster size of 50 voxels).

Compared to HS, the ED patients showed an increased cerebral activity in bilateral cerebellum, insula (BA47∖48), globus pallidus, parahippocampal gyrus (BA20), OFC (BA11) and middle cingulate cortex (MCC) (BA23), and the right putamen, superior temporal gyrus (BA21), and the left supplementary motor area (BA32, BA6) and rectus gyrus (BA11) and a decreased cerebral activity in bilateral brainstem and precuneus (BA7) and the right MCC (BA23), superior parietal lobule (BA5) and superior temporal gyrus (BA48), and the left precentral gyrus (BA6) (*P* < 0.005, a minimal cluster size of 50 voxels) (Table 2, Figure 1(a)).

### 3.2. Differences between Psychogenic ED Patients with Different TCM Pattern

#### 3.2.1. Clinical Variables

There were no significant differences in demographics (including age, height, and weight), IIEF-5 score, EHS score, SEAR score, and QEQ score between ED patients with LSSDP and patients with KDP (*P* > 0.05). There were significant differences in SDS score (30.75 ± 1.46 versus 39.99 ± 7.61) and SAS score (30.38 ± 1.77 versus 38.00 ± 6.41) between these two patterns (*P* < 0.05) (Table 3).

#### 3.2.2. Brain Activity

Compared to the patients with LSSDP, ones with KDP showed an increased cerebral activity in bilateral brainstem, cerebellum, hippocampus (left BA37, right BA27), and the right insula (BA48), thalamus, paracentral lobule (BA4), MCC (BA23), superior temporal gyrus (BA22), inferior temporal gyrus (BA20), and the left middle temporal gyrus (BA39) and a decreased cerebral activity in bilateral putamen, medial frontal gyrus (left BA10 and BA8, right BA32) and precuneus (BA7), and the right OFC (BA47), anterior cingulate cortex (ACC) (BA23), posterior cingulate cortex (PCC) (BA30), middle temporal gyrus (BA21), and the left middle frontal gyrus (BA10) and supplementary motor area (BA32) (*P* < 0.005, a minimal cluster size of 50 voxels) (Table 4, Figure 1(b)).

## 4. Discussion

This was the first neuroimaging study that focused on the differences in cerebral activity of psychogenic ED patient with different TCM patterns. The results confirmed the brain dysfunction of psychogenic ED patient compared with that of HS and preliminarily indicated that patients with different TCM patterns had relatively different cerebral activity changes.

### 4.1. Differences in Resting Brain Activity between Psychogenic ED Patients and HS

In present study, psychogenic ED patients showed abnormal cerebral activity in brainstem, cerebellum, basal ganglia, multiple limbic regions including insula, MCC, prefrontal cortex (PFC), parahippocampal gyrus, and parietal, temporal lobes (*P* < 0.005, Figure 1(a)). The results were similar to the findings in other studies on sexual dysfunction [42–45].

The limbic system is involved in emotion, reward, cognition, and human sexual activity. A number of experimental studies suggested that the limbic system play an important role in the regulation of penile erection [46, 47] and some limbic regions which expressed plentiful sex hormone receptors were essential in arousing and regulation of sex behavior [47–49]. Moreover, neuroimaging studies on human being also demonstrated the limbic regions such as amygdala, hypothalamus, and insula involved in sexual arousal [21, 38, 50, 51]. In fact, the unusual activation in PFC, CC, insula, and hippocampus can be found in nearly all reported functional brain imaging studies related to sexual psychology and sexual activity, regardless of study paradigm and analysis method [42].

In this study, we found that multiple limbic regions, including insula, MCC, PFC, and parahippocampal gyrus in psychogenic ED patients, showed significant cerebral functional changes in resting state. Other neuroimaging studies [43–45] also proved the dysfunction of limbic system in ED patients. For example, Hagemann and his coinvestigators [43] found the abnormal cerebral activities in ACC and PFC in ED patients in visual sexual stimuli task state. Cera et al. [45] found that ED patients showed decreased connectivity values in the inferior parietal lobes, medial PFC, and the right insula and increased connectivity values in the ACC. Furthermore, our previous diffusion tensor imaging (DTI) study [27] demonstrated that multiple white matter regions which associated with limbic system such as corpus callosum (genu, body, and splenium), internal capsule (anterior limb and posterior limb), and corona radiata had significantly microstructural alternations. All these studies proved that functional and structural abnormalities in limbic system might be an important character of the central pathogenesis of ED.

In this study, decreased activities in brainstem were observed in psychogenic ED patients compared with HS. Some researchers found that transition zones between the midbrain and pons, dorsal pons, and cerebral peduncles were strongly activated during sexual arousal with visual sexual stimulation, and the regional cerebral blood flow (rCBF) of midbrain was significantly increased [52]. These findings consistently suggested brainstem involved in the regulation of sex activity. Meanwhile, we found that activities in the bilateral cerebellum significantly decreased in the psychogenic ED patients compared to HS. Redouté et al. reported the positive correlation between markers of sexual arousal (perceived sexual arousal, penile tumescence) and rCBF in the vermis and the left part of the cerebellum, respectively [51]. Similarly, a cerebellar activation was found in response to excerpts of erotic films [53, 54]. In addition, meta-analysis indicated cerebellum activation consistently reported across functional imaging studies of sexual arousal [42]. Along with the previous studies, our results also suggested that cerebellum is involved in human sexual arousal and that cerebellum function abnormality is a potential central pathological character in psychogenic ED patient.

### 4.2. Differences in Resting Brain Activity between Different Patterns of Psychogenic ED Patient

In current study, our findings indicated that the cerebral activity differences between patients with KDP and LSSDP mainly are in brainstem, cerebellum, insula, CC (including ACC, MCC, and PCC), and OFC. These regions not only are involved in the central regulation of human sexual behavior, but also play important role in cognition as well as emotion modulation.

Among these regions, the brainstem, the important pathways for cerebrum, cerebellum, and spinal cord interconnection, contains the vital centers controlling the basic life activities. The cerebellum provides a significant role in both human sexual arousal [55] and emotion regulation [56, 57]. For example, Siuda et al. [58] reported that cerebellum participated in detection, integration, and filtration of emotional information and in regulation of autonomic responses. The insula, an important part of limbic system, serves as a primary “interoceptive cortex” for the integration of viscera and emotion. Several studies indicated that insula activation was specifically correlated with penile erection [24, 59] and that insula processing of feeling showed cultural effects [60]. CC, an important part of limbic system, is involved in emotion, learning, and memory. In CC, ACC and MCC are considered closely related to sexual psychology and sexual activity and have important interconnection with insula, PFC, and other subcortical structures. The OFC is seen as a central node in the emotional circuits of the brain. Profoundly altered emotion regulation is a hallmark of damage or dysfunction within OFC [61, 62]. So the current results indicated that the brain regions associated with emotion modulation were the main different brain areas between the psychogenic ED patients with KDP and the psychogenic ED patients with LSSDP.

Furthermore, this study found that the SAS score and the SDS score of the patients with LSSDP were significantly higher than those of patients with KDP. This result indicated the emotional symptoms were the main difference between LSSDP patients and KDP patients. The theory of TCM holds that the emotional disorders such as anxiety and depression are the main characteristics of liver-qi stagnation pattern [63]. Although the sample size of each pattern was relatively smaller, the results were also consisted with the theory of TCM.

So, in this study, the scores of SAS and SDS were the main different clinical variables between the psychogenic ED patients with LSSDP and the patients with KDP, and the brain regions involved in affect regulation were the main different brain areas between the two patterns. This neuroimaging result might be an objective reference for distinguishing LSSDP from KDP in pattern differentiation of TCM.

## 5. Conclusion

This study investigated the cerebral activity changes in psychogenic ED patient and explored the differences in brain function between ED patients with different patterns. The results confirmed that psychogenic ED patient had cerebral dysfunction in resting state and firstly demonstrated that the brain activity of ED patients with KDP differed from those with LSSDP.

In clinic practice, the scales for pattern differentiation are faster and more convenient than neuroimaging scan, but the results of questionnaires are subjective and are easily affected by experience of doctors. So although the sample size in our study was relatively smaller and the *P* value of clinical variables was not corrected for multiple comparisons, this study provided potentially objective evidence for pattern differentiation of ED and a new approach to further study the mechanism of TCM pattern.

## Figures and Tables

**Table 1:** Comparison between psychogenic ED patients and healthy subjects: clinical variables.

Characteristic	ED patients *N* = 27	Healthy subjects *N* = 27	*P* value
Age (y), mean ± SD	33.22 ± 5.92	31.41 ± 5.82	0.261
Weight (Kg), mean ± SD	65.11 ± 6.66	66.52 ± 9.47	0.530
Height (cm), mean ± SD	171.67 ± 3.98	171.41 ± 4.37	0.821
IIEF-5 (0–25), mean ± SD	13.56 ± 3.61	22.26 ± 0.95	0.000
EHS (1–4), mean ± SD	2.70 ± 0.54	3.93 ± 0.27	0.000
SEAR (0–80), mean ± SD	32.38 ± 11.14	68.36 ± 4.96	0.000
QEQ (0–100), mean ± SD	33.63 ± 15.44	80.04 ± 7.20	0.000

**Table 2:** Comparison between psychogenic ED patients and healthy subjects: resting brain activity.

Regions	Sign	Side	Talairach			*t* value	BA
			*X*	*Y*	*Z*		
Cerebellum	↑	R	11	−64	−50	3.40	—
	↑	L	−26	−37	−35	3.13	—
Insula	↑	R	32	23	−2	3.48	BA47
	↑	L	−32	22	−3	4.06	BA48
Globus pallidus	↑	R	20	2	8	2.66	—
	↑	L	−10	1	−1	2.75	—
Parahippocampal gyrus	↑	R	33	−25	−17	3.29	BA20
	↑	L	−25	−24	−16	3.03	BA20
OFC	↑	R	8	30	−13	3.13	BA11
	↑	L	−7	29	−12	2.68	BA11
MCC	↑	R	11	23	38	2.79	BA32
	↑	L	−7	20	36	2.81	BA32
Putamen	↑	R	23	0	8	2.56	—
Superior temporal gyrus	↑	R	44	−42	7	4.01	BA21
Rectus gyrus	↑	L	−7	30	−17	2.99	BA11
Supplementary motor area	↑	L	−10	0	67	2.72	BA6
	↑	L	−8	16	48	3.98	BA32
Brainstem	↓	R	3	−35	−30	−3.13	—
	↓	L	−1	−34	−29	−2.88	—
Precentral gyrus	↓	L	−44	2	42	−3.23	BA6
MCC	↓	R	2	−28	34	−3.40	BA23
Superior parietal lobule	↓	R	16	−44	66	−2.83	BA5
Superiortemporalgyrus	↓	R	60	−10	11	−4	BA48
Precuneus	↓	R	10	−65	37	−2.80	BA7
	↓	L	−10	−65	35	−3.05	BA7

“Sign” indicates whether the structure showed a signal increase or decrease. “↑/↓”: increase/decrease. R: right; L: left; BA: Brodmann area; OFC: orbitofrontal cortex; MCC: middle cingulate cortex; *P* < 0.005, a minimal cluster size of 50 voxels.

**Table 3:** Comparison between psychogenic ED patients with liver-qi stagnation and spleen deficiency pattern and kidney-yang deficiency pattern: clinical variables.

	Kidney-yang deficiency pattern *N* = 10	Liver-qi stagnation and spleen deficiency pattern *N* = 10	*P* value
Age (y), mean ± SD	32.90 ± 5.89	32.10 ± 3.17	0.71
Weight (Kg), mean ± SD	65.30 ± 11.10	66.90 ± 6.36	0.70
Height (cm), mean ± SD	172.00 ± 5.14	173.60 ± 4.09	0.45
IIEF-5 (0–25), mean ± SD	14.20 ± 3.85	15.00 ± 2.16	0.58
EHS (1–4), mean ± SD	2.90 ± 0.32	2.90 ± 0.32	1.00
SEAR (0–80), mean ± SD	31.41 ± 10.14	33.14 ± 7.32	0.67
QEQ (0–100), mean ± SD	33.36 ± 16.79	31.28 ± 5.29	0.71
SDS (0–100), mean ± SD	30.75 ± 1.46	39.99 ± 7.61	0.003
SAS (0–100), mean ± SD	30.38 ± 1.77	38.00 ± 6.41	0.002

**Table 4:** Comparison between psychogenic ED patients with liver-qi stagnation and spleen deficiency pattern and kidney-yang deficiency pattern: resting brain activity.

Regions	Sign	Side	Talairach			*t* value	BA
			*X*	*Y*	*Z*		
Brainstem	↑	R	8	−19	7	3.21	—
	↑	L	−7	−23	−10	3.13	—
Cerebellum	↑	R	14	−51	−31	3.54	—
	↑	L	−14	−66	−22	4.82	—
Hippocampus	↑	R	22	−30	−4	3.48	BA27
	↑	L	−31	−35	4	3.59	BA37
Insula	↑	R	39	−7	17	3.16	BA48
Thalamus	↑	R	10	−21	1	3.07	—
Paracentral lobule	↑	R	8	−33	53	3.13	BA4
MCC	↑	R	2	−15	48	2.88	BA23
Superior temporal gyrus	↑	R	57	−33	11	3.16	BA22
Inferior temporal gyrus	↑	R	59	−42	−15	4	BA20
Middle temporal gyrus	↑	L	−43	−50	20	3.4	BA39
Putamen	↓	R	24	13	−2	−2.94	—
	↓	L	−22	18	3	−3.61	—
Medial frontal gyrus	↓	R	13	57	25	−2.58	BA10
	↓	R	8	29	54	−3.48	BA8
	↓	L	−1	41	30	−2.64	BA32
Middle frontal gyrus	↓	L	−35	49	11	−4.47	BA10
Supplementary motor area	↓	L	−4	17	46	−3.59	BA6
OFC	↓	R	8	51	−4	−3.02	BA10
ACC	↓	R	11	24	29	−2.59	BA23
PCC	↓	R	4	−49	26	−2.72	BA30
Middle temporal gyrus	↓	R	51	−39	2	−3.46	BA21
Precuneus	↓	R	8	−64	33	−3.95	—
	↓	L	−3	−68	37	−2.99	BA7

“Sign” indicates whether the structure showed a signal increase or decrease. “↑/↓”: increase/decrease. R: right; L: left; BA: Brodmann area; OFC: orbitofrontal cortex; ACC: anterior cingulate cortex; MCC: middle cingulate cortex; PCC: posterior cingulate cortex.

*P* < 0.005, a minimal cluster size of 50 voxels.
